# ﻿Three new species of *Atkinsoniella* (Arthropoda, Insecta, Hemiptera, Cicadellidae, Cicadellinae) from China, with an updated checklist to the known species worldwide

**DOI:** 10.3897/zookeys.1161.101062

**Published:** 2023-05-11

**Authors:** Yan Jiang, Xiao-fei Yu, Mao-fa Yang

**Affiliations:** 1 Institute of Entomology, Guizhou Provincial Key Laboratory for Agricultural Pest Management of the Mountainous Region, Guizhou University, Guiyang 550025, China Guizhou University Guiyang China; 2 College of Tobacco Sciences, Guizhou University, Guiyang 550025, China Guizhou University Guiyang China

**Keywords:** Auchenorrhyncha, leafhopper, morphology, taxonomy, Tibet, Yunnan

## Abstract

The sharpshooter genus *Atkinsoniella* Distant, 1908 includes 99 valid species worldwide. Here, three new species from China are described and illustrated: *Atkinsoniellastenopyga*, *A.wangi*, and *A.yingjiangensis***spp. nov.** An updated checklist of the known *Atkinsoniella* species worldwide based on the data of previous literature and studied materials is also provided. All the type specimens of three new species are deposited at the Institute of Entomology, Guizhou University, Guiyang, China.

## ﻿Introduction

The genus *Atkinsoniella* is a relatively large genus of the subfamily Cicadellinae. It was established by Distant (1908) with two new species: *A.decisa* (type species) and *A.maculata*. Young (1986) systematically revised *Atkinsoniella*, proposed 15 new combinations and 13 synonyms, described 10 new species, and confirmed 26 valid species of this genus. Thereafter, new species were described successively.

Feng and Zhang (2015) provided a checklist of 75 known species worldwide and described two new species. Yang et al. (2017) conducted a systematic morphological study of 88 *Atkinsoniella* species from China, including 33 new species, two Chinese new records, 12 new synonyms, and proposed *Curvufacies* Kuoh, 1993 as a new synonym of *Atkinsoniella*. Subsequently, 2 new species from China and Pakistan were described (Naveed and Zhang 2018; Jiang et al. 2022).

To date, 99 valid species were described worldwide, of which 89 species occurred in China, and a few were scattered in Bay of Bengal, Bhutan, India, Indonesia, Kingdom of Bhutan, Laos, Malay Islands, Malaysia, Myanmar, Nepal, Pakistan, Philippines, Thailand, and Vietnam (Feng and Zhang 2015; Yang et al. 2017; Naveed and Zhang 2018; Jiang et al. 2022). In this study, the description, male genitalia and habitus photos of three new species, *A.stenopyga*, *A.wangi*, and *A.yingjiangensis* spp. nov. from Qinghai–Tibet Plateau (Tibet Autonomous Region) and Yunnan-Guizhou Plateau (Yunnan Province) of China are provided. The checklist of all known *Atkinsoniella* species worldwide and the three new species is updated.

## ﻿Material and methods

### ﻿Morphology

The specimens were collected by sweeping (27–35 sweeps per collecting event) on shrubs and weeds using 2.5 m insect sweep nets on day-light and off-set sun by using a 500W high-pressure mercury lamps; all materials were preserved in absolute ethanol and stored at -20 °C in the laboratory. The abdomens of specimens were detached and soaked in 10% NaOH solution, boiled for 1–3 min, rinsed with water to remove traces of NaOH, and transferred to glycerol for further dissection, photography and preservation. The habitus and male genitalia were photographed using a KEYENCE VHX-6000 digital camera and a Nikon Eclipse Ni-E microscope, respectively. Adobe Photoshop 2020 was used to edit compiled photos. The length of the body was measured from the vertex to the rear of the forewings using a KEYENCE VHX-6000 digital camera. The morphological terminology is adapted from Young (1968, 1986) and Yang et al. (2017). The holotype and paratypes were deposited at the Institute of Entomology, Guizhou University, Guiyang, China (**GUGC**).

## ﻿Results

### ﻿ Atkinsoniella

Taxon classificationAnimaliaHemipteraCicadellidae

Distant, 1908

3E2E5085-7F3F-59E9-B2C3-D12DAF74212F

Atkinsoniella Distant, 1908: 235.
Soibanga Distant, 1908: 236.
Curvufacies Kuoh, 1993: 38.

#### Type species.

*Atkinsonielladecisa* Distant, 1908.

#### Distribution.

Palaearctic, Oriental.

### ﻿ Atkinsoniella stenopyga

Taxon classificationAnimaliaHemipteraCicadellidae

Jiang & Yang sp. nov.

899B50EA-1F85-58FD-BB79-849F315A9FF1

https://zoobank.org/325305A3-840F-480C-B5CF-1F5FC86957C5

Figs 1A–D , 2A–F

#### Description.

Crown and thorax canary yellow and greyish white in dorsal view; a small subcircular black spot at apex of head and basal margin medially, and interocular width 2× wider than long; eyes fuscus; ocelli greyish white with narrow black border; forewing light orange; face off-white, frontoclypeus median with a broad yellowish white longitudinal; thorax and abdomen yellowish white in ventral view; legs orangish with pretarsi black or dark brown.

**Figure 1.**
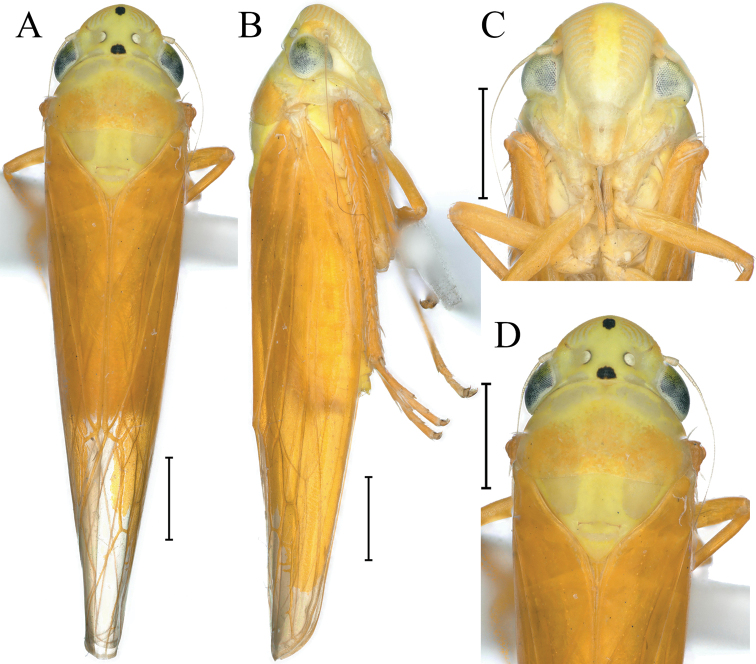
External features of *Atkinsoniellastenopyga* Jiang & Yang, sp. nov., male holotype **A** habitus, dorsal view **B** habitus, lateral view **C** face, anterior view **D** head and pronotum, dorsal view. Scale bars: 1000 μm.

Anterior margin of crown broadly rounded and convex, and median length of crown shorter than interocular width. Ocelli nearest to midline and posterior margin than eyes, lateral area concave, each ocellus further from the other than to the adjacent eye. Face with frontoclypeus flat medially; muscle impressions distinct and extend to the tip of crown; clypeal sulcus blurred in the median; anteclypeus longitudinally gibbous. Pronotum wider than head, anterior margin arcuately convex, posterior margin with medially concave. Scutellum with medial transverse depression. Forewings with distinct apical membranous area, base of second cells more proximal than third cells transversely.

**Figure 2.**
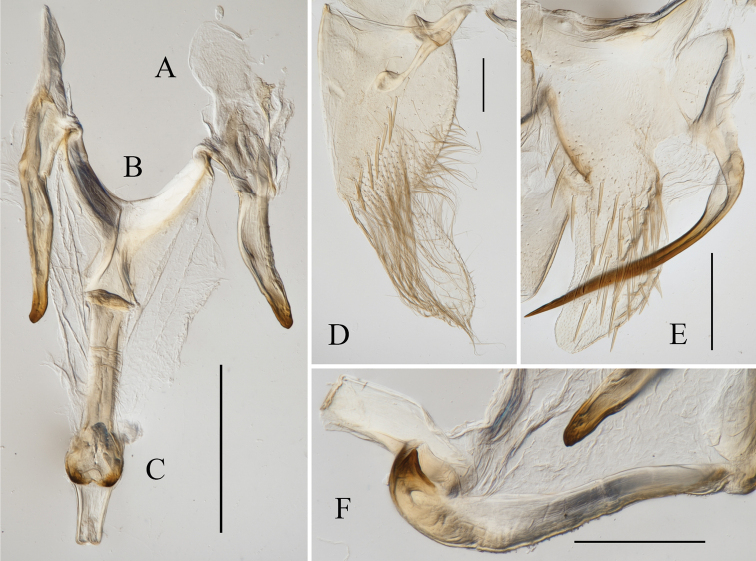
Male genitalia of *Atkinsoniellastenopyga* Jiang & Yang, sp. nov. **A** style **B** connective **C** aedeagus and paraphysis, ventral view **D** subgenital plate, ventral view **E** pygofer, lateral view **F** aedeagus and paraphysis, lateral view. Scale bars: 200 μm.

Male pygofer narrowly rounded posteriorly and convex dorsally, posterior half long scoop-shaped with macrosetae; pygofer processes slender and strongly sclerosed, base broad with microsetae; bending dorsad from basal one-third and then extending straightly, tip acute and exceeding dorsal margin posteriorly of pygofer. Subgenital plates in ventral view convex and short with one row of macrosetae uniseriate obliquely, long dense mid microsetae, and posterior half with long and short microsetae dispersedly with apex rounded. Aedeagus stubby and straight, with posterior margin truncate and dorsal margin concave subbasally, one protuberance at base ventrally articulating with paraphysis, and concave at the articulation with paraphysis apically; paraphysis long and thick, apical portion intumescent, apex bifurcated and articulating with aedeagus. Connective Y-shaped; style slender, with tip tapered and curved.

#### Etymology.

The specific epithet is the combined noun of stenos and tail from Greek, *stenopyga*, referring a narrow pygofer shape.

#### Measurement.

Length of male 7.8–8.0 mm.

#### Material examined.

***Holotype***: ♂, Motuo, Tibet, China, 18 August 2020, coll. Xian-Yi Wang. ***Paratype***, 1♂, same data as holotype.

#### Remarks.

This species is similar to *A.thaloidea* Young, 1986, *A.flavipenna* Li & Wang, 1992, *A.uniguttata* Li, 1993, and *A.bowa* Yang, Meng & Li, 2017 in appearance, but can be easily differentiated from these species by the following characteristics: (1) pygofer slender; (2) aedeagus stubby with posterior margin truncate; and (3) two pointed dentate protrusions at the apex of the paraphysis incurved dorsally and embracing.

#### Distribution.

China (Tibet).

### ﻿ Atkinsoniella wangi

Taxon classificationAnimaliaHemipteraCicadellidae

Jiang & Yang sp. nov.

15EA130B-EC1B-5D7C-94B7-F133A2B19756

https://zoobank.org/89195CDF-6EDC-4BF0-AEDB-A7508F9A9795

Figs 3A–D , 4A–F

#### Description.

Crown, thorax and forewings orange in dorsal view; crown with a black spot at anterior margin medially and a smaller black spot at posterior margin medially; eyes off-white or dark brown; ocelli off-white with black border; forewings orange, with apical membranous area darker; face, thorax and abdomen in ventral view, legs orange.

**Figure 3.**
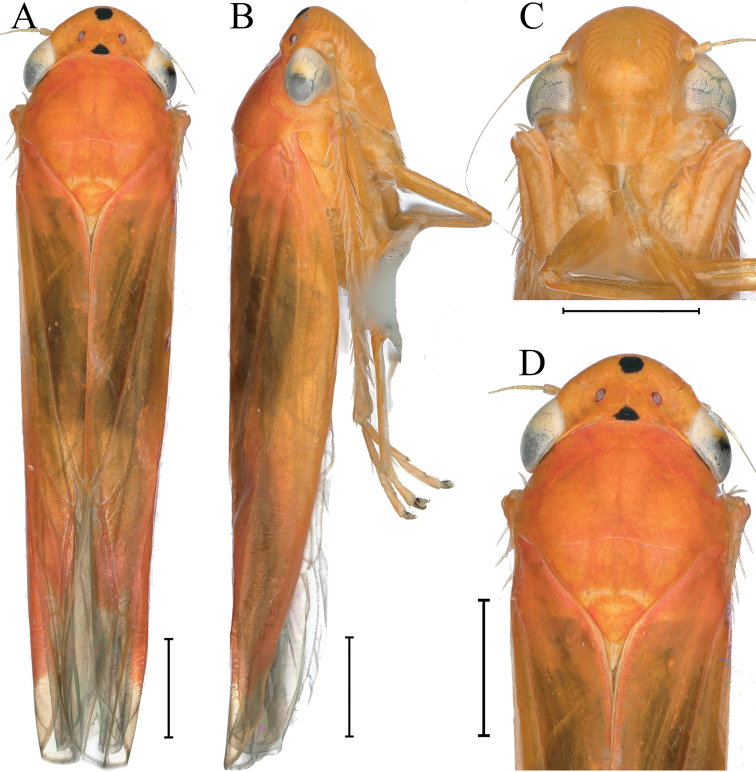
External features of *Atkinsoniellawangi* Jiang & Yang, sp. nov., male holotype **A** habitus, dorsal view **B** habitus, lateral view **C** face, anterior view **D** head and pronotum, dorsal view. Scale bars: 1000 μm.

Crown with anterior margin rounded prominently, and interocular width 2× wider than long. Ocelli nearest to midline and posterior margin than eyes, lateral area concave, each ocellus slightly further from the other one than to the adjacent eye. Face with a nodular protrusion in the center, frontoclypeus flat medially, muscle impressions distinct and extending to the tip of crown, clypeal sulcus distinct medially. Pronotum broader than head, anterior margin protruding roundly, posterior margin with medially concave. Scutellum convex anterior and posterior to transverse depression, with transverse depression arcuate, a large black spot near each basal angle in some specimens. Forewings with four apical cells, the base of the second cells more basal than third cells transversely, apical membranous area distinct.

**Figure 4.**
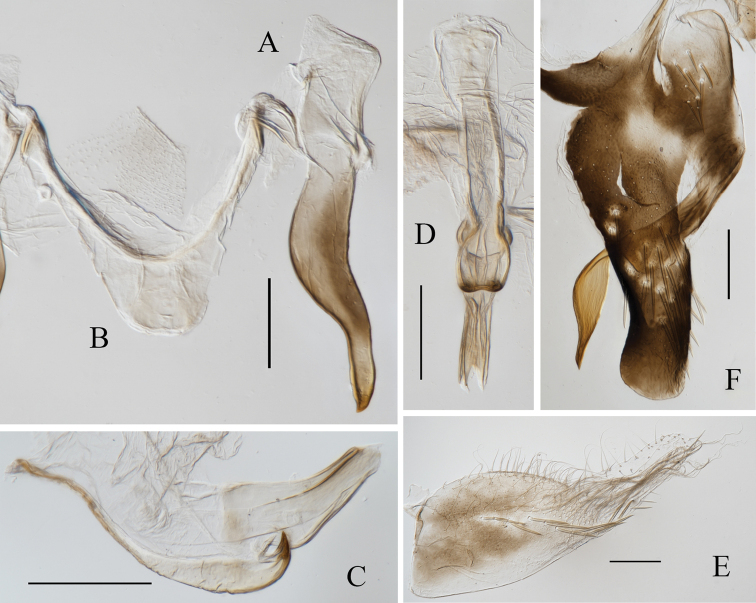
Male genitalia of *Atkinsoniellawangi* Jiang & Yang, sp. nov. **A** style **B** connective **C** aedeagus and paraphysis, lateral view **D** aedeagus and paraphysis, ventral view **E** subgenital plate, ventral view **F** pygofer, lateral view. Scale bars: 200 μm.

Male pygofer slender, posterior portion tilted dorsad and posterior margin round, posterior half with macrosetae; pygofer process lamellate, base with several macrosetae, bending dorsad from basal one-third, posterior one-third portion lamellate broadly and tortile backward and apex acute. Subgenital plates broad at base, posterior half narrow and bent dorsally, one row of macrosetae uniseriate obliquely in the median, lateral area with long dense microsetae, apical half with short microsetae dispersedly. Aedeagus broad at base, posterior half constricted and tilted dorsad, tip swordlike, ventral margin protuberant at the articulation with the tip of paraphysis and concave subbasally; paraphysis dilated apically and constricted subapically, apex bifurcated and articulating with aedeagus. Connective V-shaped. Style wide at base and tapered at tip, apex acute.

#### Etymology.

The new species is named after the family name of collector Xian-Yi Wang.

#### Measurement.

Length of male 7.9–8.0 mm.

#### Material examined.

***Holotype***: ♂, Tongmai, Tibet, China, 18 August 2020, coll. Xian-Yi Wang. ***Paratypes***, 1♂ (light trapped) +11♂♂, same data as holotype; 1♂, Tongmai, Tibet, China, 19 August 2020, light trapped, coll. Xian-Yi Wang.

#### Remarks.

This species is similar to *A.curvata* Zhang & Kuoh in appearance and male genitalia, but can be distinguished from the latter by the following characteristics: (1) crown with a black spot in the median of the anterior margin, and the smaller black spot at the basal margin medially narrower than the width between the ocelli, but the latter only with a large V-shaped black spot below the ocelli; (2) pygofer of the new species slender overall, with posterior margin rounded, while pygofer of the latter slender at apical one-third, with the posterior margin truncate; (3) aedeagus of the new species tilted dorsally at the posterior half, while aedeagus of the latter straight overall; and (4) connective of the new species V-shaped, but connective of the latter Y-shaped.

#### Distribution.

China (Tibet).

### ﻿ Atkinsoniella yingjiangensis

Taxon classificationAnimaliaHemipteraCicadellidae

Jiang & Yang sp. nov.

C2F1BBF5-AAE6-5C59-9787-451AE04E75A4

https://zoobank.org/6FD2EA20-B2DA-442B-B50E-A35F91051243

Figs 5A–D , 6A–G

#### Description.

Head and thorax dark orange in dorsal view; crown with a black spot at the anterior margin medially, basal margin with a black spot below each ocellus, and lateral margin with a black spot anterior to each antennal ledge; eyes dark brown; ocelli gray; pronotum with two small black spots abreast in the center, and posterior area with two large black spots transversely; scutellum with a large triangular black spot at each basal, and the base of the two spots linked; forewings black, with three longitudinal grayish white stripes, apical membranous area dark brown; face dark orange; thorax black in ventral view; legs grayish white.

**Figure 5.**
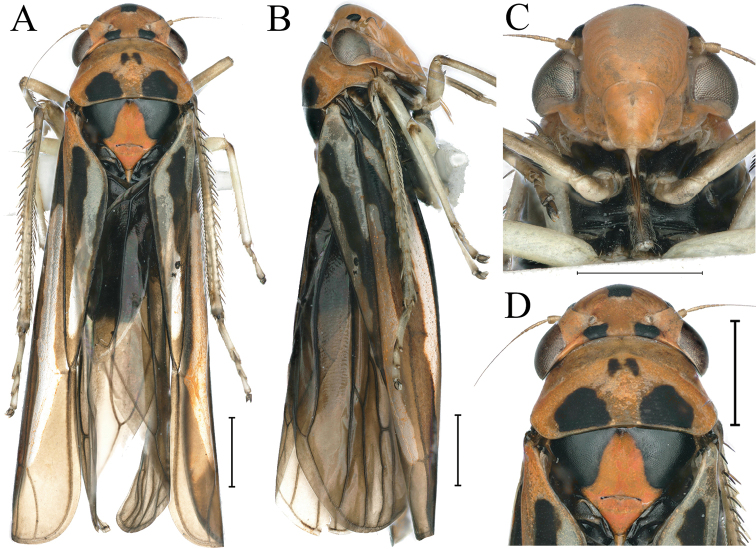
External features of *Atkinsoniellayingjiangensis* Jiang & Yang, sp. nov., male holotype **A** habitus, dorsal view **B** habitus, lateral view **C** face, anterior view **D** head and pronotum, dorsal view. Scale bars: 1000 μm.

Crown with anterior margin convex roundly, median length of crown approximately equal to half of interocular width, concave lateral area of ocelli. Ocelli located at the line of anterior eyes, each ocellus slightly further from the other one than to the adjacent eye. Face with frontoclypeus flat in the median, muscle impressions and clypeal sulcus distinct. Pronotum wider than head, anterior margin convex roundly and posterior margin concave. Scutellum with transverse depression slightly posterior to the median. Forewings with apical membranous area not obvious, base of the second and third cells almost aligned transversely.

**Figure 6.**
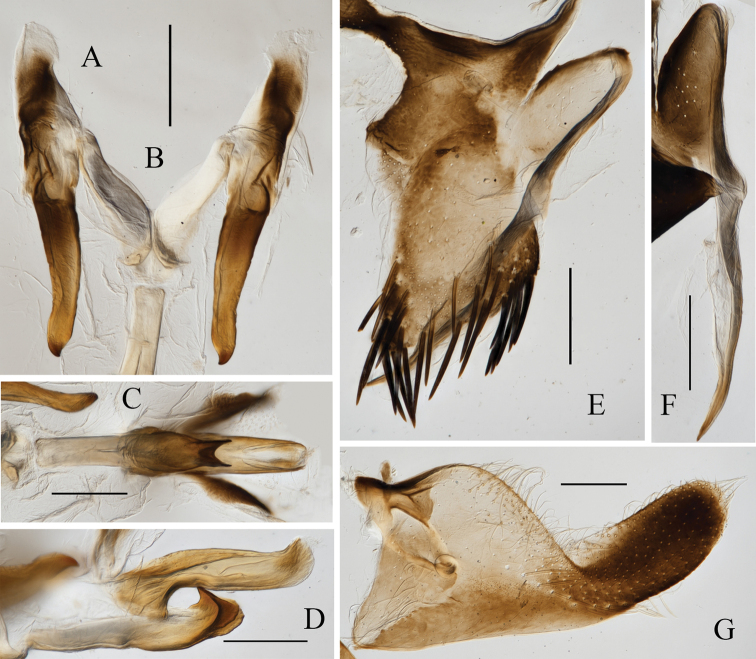
Male genitalia of *Atkinsoniellayingjiangensis* Jiang & Yang, sp. nov. **A** style **B** connective **C** aedeagus and paraphysis, ventral view **D** aedeagus and paraphysis, lateral view **E** pygofer, lateral view **F** pygofer process, lateral view **G** subgenital plate, ventral view; Scale bars: 200 μm.

Male pygofer narrowly rounded posteriorly, tip oblique dorsally, dorsal margin with lamellar prominence at basal one-third, with macrosetae in posterior half and microsetae in the median dispersedly; pygofer processes with microsetae at base, arising basiventrally on each side and extending dorsolateral posteriorly of pygofer, apex acute and exceeding posterior margin of pygofer. Subgenital plates broad at base and constrictive at tip, with one row of macrosetae uniseriate obliquely, long microsetae on lateral area, short microsetae on posterior half. Aedeagus slender, bent dorsally at tip and curved ventral, ventral margin with a horned protuberance basically articulating with paraphysis; paraphysis wide subapically, with two lamellate bulges at tip, apex uncinate and articulating with aedeagus. Connective Y-shaped. Style narrow at posterior portion, apex curved and exceeding the tip of connective.

#### Etymology.

The name of the new species is derived from Yingjiang where the type specimens were collected.

#### Measurement.

Length of male 8.0–8.1 mm.

#### Material examined.

***Holotype***: ♂, Yingjiang, Yunnan, China, 25 June 2019, coll. Tie-Long Xu. ***Paratypes***, 2♂♂, same data as holotype.

#### Remarks.

This species is similar to *A.limba* Kuoh, 1991 in male genitalia, but markedly differ in the following characteristics: (1) pronotum with two small black spots abreast in the center and two large black spots transversely at posterior area; (2) apex of the pygofer process exceeding the posterior margin of the pygofer; (3) aedeagus concave obviously at the ventral margin basally; and (4) paraphysis inflated subapically and posterior margin Λ-shaped in ventral view.

#### Distribution.

China (Yunnan).

##### ﻿Checklist of the genus *Atkinsoniella* worldwide (updated from Yang et al. 2017)

***Atkinsoniellaalbimacula* Yang & Li, 2002**

*Atkinsoniellaalbimacula* Yang & Li, 2002a: 556.

**Distribution.** China (Yunnan).

***Atkinsoniellaalbipenna* Yang, Meng & Li, 2017**

*Atkinsoniellaalbipenna* Yang, Meng & Li, 2017: 219.

**Distribution.** China (Guangxi, Yunnan).

***Atkinsoniellaalcmena* (Distant, 1908)**

*Tettigoniellaalcmena* Distant, 1908: 219.

*Atkinsoniellaalcmena* (Distant): Young, 1986: 96.

**Distribution.** China (Tibet), India.

***Atkinsoniellaalternata* Young, 1986**

*Atkinsoniellaalternata* Young, 1986: 100.

**Distribution.** China (Guizhou, Taiwan, Yunnan).

***Atkinsoniellaangula* Kuoh, 1992**

*Atkinsoniellaangula* Kuoh, 1992: 126.

**Distribution.** China (Gansu, Yunnan).

***Atkinsoniellaanabella* Young, 1986**

*Atkinsoniellaanabella* Young, 1986: 107.

**Distribution.** Bhutan, India, Nepal.

***Atkinsoniellaatrata* Yang, Meng & Li, 2017**

*Atkinsoniellaatrata* Yang, Meng & Li, 2017: 207.

**Distribution.** China (Yunnan).

***Atkinsoniellaatronotata* (Distant, 1918)**

*Kollaatronotata* Distant, 1918: 10.

*Atkinsoniellaatronotata* (Distant): Young, 1986: 96.

**Distribution.** India.

***Atkinsoniellaaurantiaca* Cai & Kuoh, 1995**

*Atkinsoniellaaurantiaca* Cai & Kuoh, 1995: 89.

**Distribution.** China (Guizhou, Hubei, Jiangxi, Yunnan, Zhejiang).

***Atkinsoniellabeaka* Yang, Meng & Li, 2017**

*Atkinsoniellabeaka* Yang, Meng & Li, 2017: 218.

**Distribution.** China (Yunnan).

***Atkinsoniellabella* (Walker, 1851)**

*Tettigoniabella* Walker, 1851: 778.

*Atkinsoniellabella* (Walker): Young, 1986: 96.

**Distribution.** India, Nepal.

***Atkinsoniellabiundulata* Meng, Yang & Ni, 2010**

*Atkinsoniellabiundulata* Meng, Yang & Ni, 2010: 42.

**Distribution.** China (Yunnan).

***Atkinsoniellabowa* Yang, Meng & Li, 2017**

*Atkinsoniellabowa* Yang, Meng & Li, 2017: 206.

**Distribution.** China (Yunnan).

***Atkinsoniellabrevistyla* Yang & Li, 2004**

*Atkinsoniellabrevistyla* Yang & Li, 2004: 757.

**Distribution.** China (Tibet, Yunnan).

***Atkinsoniellachangae* Yang, Meng & Li, 2017**

*Atkinsoniellachangae* Yang, Meng & Li, 2017: 240.

**Distribution.** China (Yunnan).

***Atkinsoniellacontrariuscula* (Jacobi, 1944)**

*Cicadellacontrariuscula* Jacobi, 1944: 44.

*Atkinsoniellacontrariuscula* (Jacobi): Young, 1986: 97.

*Atkinsoniellafurcata* Zhang & Kuoh, 1993: 15.

**Distribution.** China (Anhui, Fujian, Guangdong, Guangxi, Guizhou, Sichuan).

***Atkinsoniellacurvata* Zhang & Kuoh, 1993**

*Atkinsoniellacurvata* Zhang & Kuoh, 1993: 13.

**Distribution.** China (Tibet, Yunnan).

***Atkinsoniellacuspidata* Meng, Yang & Ni, 2010**

*Atkinsoniellacuspidata* Meng, Yang & Ni, 2010: 45.

**Distribution.** China (Yunnan).

***Atkinsoniellacyclops* (Melichar, 1914)**

*Tettigoniellacyclops* Melichar, 1914: 127.

*Atkinsoniellacyclops* (Melichar): Young, 1986: 97.

**Distribution.** China (Hainan, Yunnan), Indonesia, Nepal.

***Atkinsonielladactylia* Yang & Li, 2000**

*Atkinsonielladactylia* Yang & Li, 2000: 410.

*Atkinsoniellatrinotata* Cai & He, 2002: 147.

**Distribution.** China (Fujian, Guangdong, Guangxi, Guizhou, Hainan, Jiangxi, Yunnan).

***Atkinsonielladecisa* Distant, 1908**

*Atkinsonielladecisa* Distant, 1908: 236.

**Distribution.** India.

***Atkinsonielladivaricata* Yang, Meng & Li, 2017**

*Atkinsonielladivaricata* Yang, Meng & Li, 2017: 205.

**Distribution.** China (Guangdong, Guangxi, Guizhou).

***Atkinsonielladormana* Li, 1992**

*Atkinsonielladormana* Li, 1992: 345.

**Distribution.** China (Chongqing, Fujian, Guizhou, Hubei, Jiangxi, Sichuan, Shaanxi, Yunnan).

***Atkinsonielladubia* Young, 1986**

*Atkinsonielladubia* Young, 1986: 104.

**Distribution.** China (Tibet), Bhutan.

***Atkinsonielladuna* Yang, Meng & Li, 2017**

*Atkinsonielladuna* Yang, Meng & Li, 2017: 236.

**Distribution.** China (Guizhou, Yunnan).

***Atkinsoniellaexpanda* Yang, Meng & Li, 2017**

*Atkinsoniellaexpanda* Yang, Meng & Li, 2017: 214.

**Distribution.** China (Yunnan).

***Atkinsoniellafishtaila* Yang, Meng & Li, 2017**

*Atkinsoniellafishtaila* Yang, Meng & Li, 2017: 221.

**Distribution.** China (Hubei).

***Atkinsoniellafistular* Naveed & Zhang, 2018**

*Atkinsoniellafistular* Naveed & Zhang, 2018: 286.

**Distribution.** Pakistan.

***Atkinsoniellaflavilega* Yang, Meng & Li, 2017**

*Atkinsoniellaflavilega* Yang, Meng & Li, 2017: 242.

**Distribution.** China (Yunnan).

***Atkinsoniellaflavipenna* Li & Wang, 1992**

*Atkinsoniellaflavipenna* Li & Wang, 1992: 95.

**Distribution.** China (Fujian, Hubei, Hunan, Guangdong, Guangxi, Guizhou, Sichuan).

***Atkinsoniellaflexa* Kuoh, 1992**

*Atkinsoniellaflexa* Kuoh, 1992: 127.

**Distribution.** China (Yunnan).

***Atkinsoniellafuripygofera* Yang & Meng, 2011**

*Atkinsoniellafuripygofera* Yang & Meng in Yang, Meng & Li, 2011: 765.

**Distribution.** China (Yunnan).

***Atkinsoniellafuscopenna* Yang & Li, 2004**

*Atkinsoniellafuscopenna* Yang & Li, 2004: 756.

**Distribution.** China (Tibet).

***Atkinsoniellagoosenecka* Yang, Meng & Li, 2017**

*Atkinsoniellagoosenecka* Yang, Meng & Li, 2017: 222.

**Distribution.** China (Chongqing, Sichuan).

***Atkinsoniellagrahami* Young, 1986**

*Atkinsoniellagrahami* Young, 1986: 105.

*Atkinsoniellanigroscuta* Zhang & Kuoh, 1993: 11.

*Atkinsoniellafurcula* Yang & Li, 2002b: 40.

**Distribution.** China (Chongqing, Gansu, Guangdong, Guizhou, Hainan, Henan, Hubei, Hunan, Shaanxi, Sichuan, Yunnan).

***Atkinsoniellagregalis* (Distant, 1908)**

*Kollagregalis* Distant, 1908: 226.

*Atkinsoniellagregalis* (Distant): Young, 1986: 97.

**Distribution.** India, Myanmar.

***Atkinsoniellaguttata* Kuoh, 1992**

*Atkinsoniellaguttata* Kuoh, 1992: 125.

**Distribution.** China (Yunnan, Tibet).

***Atkinsoniellaheae* Yang, Meng & Li, 2017**

*Atkinsoniellaheae* Yang, Meng & Li, 2017: 238.

**Distribution.** China (Tibet).

***Atkinsoniellaheiyuana* Li, 1992**

*Atkinsoniellaheiyuana* Li, 1992: 348.

*Atkinsoniellarubra* Kuoh & Cai in Cai & Kuoh, 1994: 14.

**Distribution.** China (Chongqing, Gansu, Fujian, Guangdong, Guangxi, Guizhou, Hainan, Hubei, Hunan, Jiangxi, Shaanxi, Sichuan, Tibet, Yunnan), Vietnam.

***Atkinsoniellahuangi* Yang & Zhang, 2000**

*Atkinsoniellahuangi* Yang & Zhang, 2000: 187.

**Distribution.** China (Guizhou, Sichuan, Yunnan).

***Atkinsoniellahupehna* Young, 1986**

*Atkinsoniellahupehna* Young, 1986: 118.

*Atkinsoniellaobliqua* Zhang & Kuoh, 1993: 14.

**Distribution.** China (Chongqing, Fujian, Guangxi, Guizhou, Hubei, Jiangxi, Shaanxi, Zhejiang).

***Atkinsoniellainsignata* (Haupt, 1924)**

*Tettigoniatrilineata* Melichar, 1902: 132.

*Tettigoniellatrilineatainsignata* Haupt, 1924: 306.

*Tettigellachinensis* Metcalf, 1955: 264.

*Atkinsoniellainsignata* (Haupt): Young, 1986: 97.

*Atkinsoniellalonginotata* Kuoh, 1992: 124.

**Distribution.** China (Hubei, Qinghai, Sichuan, Yunnan).

***Atkinsoniellajavana* (Melichar, 1914)**

*Kollajavana* Melichar, 1914: 124.

*Atkinsoniellajavana* (Melichar): Young, 1986: 97.

**Distribution.** Indonesia.

***Atkinsoniellajini* Yang, Meng & Li, 2017**

*Atkinsoniellajini* Yang, Meng & Li, 2017: 199.

**Distribution.** China (Tibet).

***Atkinsoniellalatior* Young, 1986**

*Atkinsoniellalatior* Young, 1986: 113.

**Distribution.** China (Guangdong, Guangxi, Hubei, Jiangxi).

***Atkinsoniellalii* Yang & Zhang, 2000**

*Atkinsoniellalii* Yang & Zhang, 2000: 186.

**Distribution.** China (Yunnan).

***Atkinsoniellalimba* Kuoh, 1991**

*Atkinsoniellalimba* Kuoh, 1991: 20.

**Distribution.** China (Fujian).

***Atkinsoniellaliui* Yang, Meng & Li, 2017**

*Atkinsoniellaliui* Yang, Meng & Li, 2017: 241.

**Distribution.** China (Tibet).

***Atkinsoniellalonga* Yang, Meng & Li, 2017**

*Atkinsoniellalonga* Yang, Meng & Li, 2017: 212.

**Distribution.** China (Yunnan).

***Atkinsoniellalongiaurita* Yang, Meng & Li, 2017**

*Atkinsoniellalongiaurita* Yang, Meng & Li, 2017: 202.

**Distribution.** China (Yunnan).

***Atkinsoniellalongiuscula* Feng & Zhang, 2015**

*Atkinsoniellalongiuscula* Feng & Zhang, 2015: 281.

**Distribution.** China (Sichuan, Yunnan).

***Atkinsoniellamalaisei* Young, 1986**

*Atkinsoniellamalaisei* Young, 1986: 102.

**Distribution.** China (Yunnan), Myanmar.

***Atkinsoniellamediofasciola* Yang & Li, 2002**

*Atkinsoniellamediofasciola* Yang & Li, 2002b: 40.

**Distribution.** China (Chongqing, Fujian, Guangxi, Sichuan).

***Atkinsoniellamembrana* Yang, Meng & Li, 2017**

*Atkinsoniellamembrana* Yang, Meng & Li, 2017: 213.

**Distribution.** China (Yunnan).

***Atkinsoniellamotuoensis* Meng, Yang & Ni, 2010**

*Atkinsoniellamotuoensis* Meng, Yang & Ni, 2010: 47.

**Distribution.** China (Tibet).

***Atkinsoniellamultiseta* Yang, Meng & Li, 2017**

*Atkinsoniellamultiseta* Yang, Meng & Li, 2017: 226.

**Distribution.** China (Yunnan).

***Atkinsoniellamungphuensis* (Distant, 1908)**

*Kollamungphuensis* Distant, 1908: 225.

*Atkinsoniellamungphuensis* (Distant): Young, 1986: 97.

**Distribution.** India, Myanmar.

***Atkinsoniellastenopyga* Jiang & Yang, sp. nov.**

**Distribution.** China (Tibet).

***Atkinsoniellanigra* Kuoh & Cai, 1994**

*Atkinsoniellanigra* Kuoh & Cai in Cai & Kuoh, 1994: 13.

**Distribution.** China (Yunnan).

***Atkinsoniellanigricephala* Li, 1992**

*Atkinsoniellanigricephala* Li, 1992: 349.

**Distribution.** China (Guizhou, Hubei, Zhejiang).

***Atkinsoniellanigridorsum* Kuoh & Zhuo, 1996**

*Atkinsoniellanigridorsum* Kuoh & Zhuo, 1996: 2.

**Distribution.** China (Chongqing, Fujian, Guizhou, Hubei, Sichuan, Zhejiang).

***Atkinsoniellanigripennis* Yang & Li, 1999**

*Atkinsoniellanigripennis* Yang & Li, 1999: 2.

**Distribution.** China (Yunnan).

***Atkinsoniellanigriscens* Yang & Li, 2004**

*Atkinsoniellanigriscens* Yang & Li, 2004: 756.

**Distribution.** China (Yunnan, Tibet).

***Atkinsoniellanigrisigna* Li, 1992**

*Atkinsoniellanigrisigna* Li, 1992: 344.

*Atkinsoniellachloritta* Yang & Li, 2002a: 558.

**Distribution.** China (Chongqing, Guangxi, Guizhou, Hubei, Sichuan, Yunnan).

***Atkinsoniellanigrita* Zhang & Kuoh, 1993**

*Atkinsoniellanigrita* Zhang & Kuoh, 1993: 12.

*Atkinsoniellabimanculata* Cai & Shen, 1998: 43.

**Distribution.** China (Chongqing, Gansu, Henan, Hubei, Sichuan, Shaanxi, Zhejiang).

***Atkinsoniellanigrominiatula* (Jacobi, 1944)**

*Cicadellanigrominiatula* Jacobi, 1944: 44.

*Atkinsoniellanigrominiatula* (Jacobi): Young, 1986: 97.

**Distribution.** China (Chongqing, Fujian, Gansu, Guangxi, Guizhou, Hubei, Jiangxi, Sichuan, Zhejiang).

***Atkinsoniellanigrosteaka* Li & Wang, 1994**

*Atkinsoniellanigrosteaka* Li & Wang, 1994: 27.

**Distribution.** China (Tibet).

***Atkinsoniellaopponens* (Walker, 1851)**

*Tettigoniaopponens* Walker, 1851: 757.

*Tettigoniellabellona* Distant, 1908: 212.

*Tettigoniellamarpessa* Distant, 1908: 215.

*Kollacanidia* Distant, 1908: 226.

*Kollamaculifrons* Schmidt, 1911: 295.

*Kollamaculifronssimilis* Schmidt, 1911: 296.

*Kollatrimaculata* Schmidt, 1911: 297.

*Tettigoniellacuprea* Melichar, 1914: 128.

*Kollatigrina* Distant, 1918: 9.

*Kollamelichari* China, 1935: 305.

*Atkinsoniellaopponens* (Walker): Young, 1986: 97.

*Atkinsoniellatriguttata* Zhang & Kuoh, 1993: 9.

**Distribution.** China (Chongqing, Fujian, Guangdong, Guangxi, Guizhou, Hainan, Jiangxi, Sichuan, Yunnan), India, Indonesia, Laos, Malay Islands, Malaysia, Myanmar, Nepal, Pakistan, Philippines, Thailand, Vietnam.

***Atkinsoniellapeaka* Yang, Meng & Li, 2017**

*Atkinsoniellapeaka* Yang, Meng & Li, 2017: 237.

**Distribution.** China (Jiangxi).

***Atkinsoniellapunica* Yang & Li, 2002**

*Atkinsoniellapunica* Yang & Li, 2002a: 556.

**Distribution.** China (Yunnan).

***Atkinsoniellarecta* Yang, Meng & Li, 2017**

*Atkinsoniellarecta* Yang, Meng & Li, 2017: 209.

**Distribution.** China (Yunnan).

***Atkinsoniellarectangulata* Yang, Meng & Li, 2017**

*Atkinsoniellarectangulata* Yang, Meng & Li, 2017: 210.

**Distribution.** China (Yunnan).

***Atkinsoniellarhomboida* Yang, Meng & Li, 2017**

*Atkinsoniellarhomboida* Yang, Meng & Li, 2017: 216.

**Distribution.** China (Yunnan).

***Atkinsoniellarinkihonis* (Matsumura, 1912)**

*Tettigoniarinkihonis* Matsumura, 1912: 36.

*Atkinsoniellarinkihonis* (Matsumura): Young, 1986: 97.

*Curvufaciessordidula* Kuoh, 1993: 39.

*Atkinsoniellatylata* Yang & Li, 1999: 1.

**Distribution.** China (Fujian, Guangxi, Guizhou, Jiangxi, Sichuan, Taiwan).

***Atkinsoniellarubrostriata* Kuoh, 1992**

*Atkinsoniellarubrostriata* Kuoh, 1992: 123.

**Distribution.** China (Yunnan).

***Atkinsoniellarufistigma* Yang, Meng & Li, 2017**

*Atkinsoniellarufistigma* Yang, Meng & Li, 2017: 233.

**Distribution.** China (Yunnan).

***Atkinsoniellasteelei* Young, 1986**

*Atkinsoniellasteelei* Young, 1986: 109.

**Distribution.** India.

***Atkinsoniellasulphurata* (Distant, 1908)**

*Tettigoniellasulphurata* Distant, 1908: 216.

*Atkinsoniellamaculata* Distant, 1908: 236.

*Bhandaratetraspila* Jacobi, 1944: 41.

*Atkinsoniellasulphurata* (Distant): Young, 1986: 97.

*Atkinsoniellatetramaculata* Zhang & Kuoh, 1993: 7.

*Atkinsoniellastigma* Zhang & Kuoh, 1993: 8.

**Distribution.** China (Chongqing, Fujian, Guangxi, Guizhou, Hubei, Hunan, Sichuan, Yunnan, Zhejiang), India, Indonesia, Myanmar.

***Atkinsoniellathalia* (Distant, 1918)**

*Tettigoniellathalia* Distant, 1918: 2.

*Atkinsoniellathalia* (Distant): Young, 1986: 97.

*Atkinsoniellarubrivenosa* Kuoh & Zhuo, 1996: 3.

**Distribution.** China (Anhui, Chongqing, Fujian, Gansu, Guangdong, Guangxi, Guizhou, Hainan, Hebei, Henan, Hubei, Hunan, Jiangxi, Shaanxi, Sichuan, Tibet, Yunnan, Zhejiang), Bay of Bengal, India, Myanmar, Pakistan, Thailand.

***Atkinsoniellathaloidea* Young, 1986**

*Atkinsoniellathaloidea* Young, 1986: 117.

**Distribution.** China (Guangdong, Guangxi, Guizhou, Hainan, Tibet, Yunnan), Myanmar.

***Atkinsoniellatiani* Yang, Meng & Li, 2017**

*Atkinsoniellatiani* Yang, Meng & Li, 2017: 230.

**Distribution.** China (Yunnan).

***Atkinsoniellatransifasciata* Yang, Meng & Li, 2017**

*Atkinsoniellatransifasciata* Yang, Meng & Li, 2017: 215.

**Distribution.** China (Yunnan).

***Atkinsoniellatridentata* Yang & Li, 2011**

*Atkinsoniellatridentata* Yang & Li in Yang, Meng & Li, 2011: 766.

**Distribution.** China (Yunnan).

***Atkinsoniellatripunctata* Dmitriev, 2020**

= *Atkinsoniellatrimaculata* Li, 1992: 347.

*Atkinsoniellatripunctata* Dmitriev, 2020: 7.

**Distribution.** China (Guizhou, Sichuan, Yunnan).

***Atkinsoniellatuberostyla* Yang, Meng & Li, 2017**

*Atkinsoniellatuberostyla* Yang, Meng & Li, 2017: 225.

**Distribution.** China (Yunnan).

***Atkinsoniellauniguttata* Li, 1993**

*Atkinsoniellauniguttata* Li, 1993: 40.

*Atkinsoniellavalida* Feng & Zhang, 2015: 283.

**Distribution.** China (Fujian, Guangxi, Guizhou, Hainan, Yunnan).

***Atkinsoniellavariata* Young, 1986**

*Atkinsoniellavariata* Young, 1986: 110.

**Distribution.** China (Guizhou, Sichuan, Tibet, Yunnan), Nepal.

***Atkinsoniellavesta* (Distant, 1908)**

*Kollavesta* Distant, 1908: 224.

*Atkinsoniellavesta* (Distant): Young, 1986: 97.

**Distribution.** India, Pakistan.

***Atkinsoniellawangi* Jiang & Yang, sp. nov.**

**Distribution.** China (Tibet).

***Atkinsoniellawarpa* Yang, Meng & Li, 2017**

*Atkinsoniellawarpa* Yang, Meng & Li, 2017: 203.

**Distribution.** China (Tibet, Yunnan).

***Atkinsoniellawui* Yang, Meng & Li, 2017**

*Atkinsoniellawui* Yang, Meng & Li, 2017: 200.

**Distribution.** China (Tibet, Yunnan).

***Atkinsoniellaxanthoabdomena* Yang, Meng & Li, 2017**

*Atkinsoniellaxanthoabdomena* Yang, Meng & Li, 2017: 232.

**Distribution.** China (Yunnan).

***Atkinsoniellaxanthonota* Kuoh, 1994**

*Atkinsoniellaxanthonota* Kuoh in Cai & Kuoh, 1994: 12.

**Distribution.** China (Yunnan).

***Atkinsoniellaxanthovena* Yang & Li, 2002**

*Atkinsoniellaxanthovena* Yang & Li, 2002c: 176.

**Distribution.** China (Jiangxi, Fujian, Hainan, Guangxi, Guizhou, Yunnan).

***Atkinsoniellaxanthovitta* Kuoh, 1994**

*Atkinsoniellaxanthovitta* Kuoh in Cai & Kuoh, 1994: 11.

**Distribution.** China (Yunnan).

***Atkinsoniellaxinfengi* Yang, Meng & Li, 2017**

*Atkinsoniellaxinfengi* Yang, Meng & Li, 2017: 223.

**Distribution.** China (Yunnan).

***Atkinsoniellayani* Yang, Meng & Li, 2017**

*Atkinsoniellayani* Yang, Meng & Li, 2017: 235.

**Distribution.** China (Yunnan).

***Atkinsoniellayingjiangensis* Jiang & Yang, sp. nov.**

**Distribution.** China (Yunnan).

***Atkinsoniellayunnanana* Yang, Meng & Li, 2017**

*Atkinsoniellayunnanana* Yang, Meng & Li, 2017: 229.

**Distribution.** China (Yunnan).

***Atkinsoniellazaihuai* Yang & Meng, 2011**

*Atkinsoniellazaihuai* Yang & Meng in Yang, Meng & Li, 2011: 766.

**Distribution.** China (Yunnan).

***Atkinsoniellazhangmuensis* Yang, Meng & Li, 2017**

*Atkinsoniellazhangmuensis* Yang, Meng & Li, 2017: 227.

**Distribution.** China (Tibet).

***Atkinsoniellazizhongi* Jiang & Yang, 2022**

*Atkinsoniellazizhongi* Jiang & Yang in Jiang, Li, Yu & Yang, 2022: 5

**Distribution.** China (Hubei, Guizhou, Zhejiang).

## Supplementary Material

XML Treatment for Atkinsoniella

XML Treatment for Atkinsoniella stenopyga

XML Treatment for Atkinsoniella wangi

XML Treatment for Atkinsoniella yingjiangensis
